# A microelectromechanical system artificial basilar membrane based on a piezoelectric cantilever array and its characterization using an animal model

**DOI:** 10.1038/srep12447

**Published:** 2015-07-31

**Authors:** Jongmoon Jang, JangWoo Lee, Seongyong Woo, David J. Sly, Luke J. Campbell, Jin-Ho Cho, Stephen J. O’Leary, Min-Hyun Park, Sungmin Han, Ji-Wong Choi, Jeong Hun Jang, Hongsoo Choi

**Affiliations:** 1 Daegu Gyeongbuk Institute of Science and Technology (DGIST), Department of Robotics Engineering, Daegu, 711-873, South Korea; 2 DGIST, DGIST-ETH Microrobot Research Center, Daegu, 711-873, South Korea; 3 Kyungpook National University, Graduate School of Electrical Engineering and Computer Science, Daegu, 702-701, South Korea; 4 The University of Melbourne, Department of Otolaryngology, Melbourne, Victoria 3002, Australia; 5 Boramae Medical Center, Seoul Metropolitan Government - Seoul National University, Department of Otorhinolaryngology, Seoul, 156-707, South Korea; 6 DGIST, Department of Information & Communication Engineering, Daegu, 711-873, South Korea; 7 Kyungpook National University School of Medicine, Department of Otorhinolaryngology-Head and Neck Surgery, Daegu, 700-721, South Korea; 8 Kyungpook National University Hospital, Daegu, 700-721, South Korea

## Abstract

We proposed a piezoelectric artificial basilar membrane (ABM) composed of a microelectromechanical system cantilever array. The ABM mimics the tonotopy of the cochlea: frequency selectivity and mechanoelectric transduction. The fabricated ABM exhibits a clear tonotopy in an audible frequency range (2.92–12.6 kHz). Also, an animal model was used to verify the characteristics of the ABM as a front end for potential cochlear implant applications. For this, a signal processor was used to convert the piezoelectric output from the ABM to an electrical stimulus for auditory neurons. The electrical stimulus for auditory neurons was delivered through an implanted intra-cochlear electrode array. The amplitude of the electrical stimulus was modulated in the range of 0.15 to 3.5 V with incoming sound pressure levels (SPL) of 70.1 to 94.8 dB SPL. The electrical stimulus was used to elicit an electrically evoked auditory brainstem response (EABR) from deafened guinea pigs. EABRs were successfully measured and their magnitude increased upon application of acoustic stimuli from 75 to 95 dB SPL. The frequency selectivity of the ABM was estimated by measuring the magnitude of EABRs while applying sound pressure at the resonance and off-resonance frequencies of the corresponding cantilever of the selected channel. In this study, we demonstrated a novel piezoelectric ABM and verified its characteristics by measuring EABRs.

The cochlea is a key organ for sound perception in the mammalian auditory system. It is a transducer that converts incoming acoustic pressure into bioelectric signals that stimulate auditory neurons^1^,^2^. In addition to transduction, the cochlea has a frequency selectivity function due to the varying rigidity of the basilar membrane (BM)^3^,^4^. The BM is a flexible membrane that responds to sounds of different frequencies according to its width, thickness, and stiffness. The basal region responds to high-frequency sounds, while the apical region reacts to low-frequency sounds. The human cochlea operates over a frequency band from 20 Hz to 20 kHz, covers a 120-dB dynamic range and can separate an incoming sound wave into ca. 3,500 channels of frequency information^1^,^5^. Additional frequency selectivity and sensitivity to low-level sounds is provided by active feedback from the cochlear amplifier.

Von Békésy investigated the function of the cochlea and constructed a mechanical model^6^,^7^. At present, several research groups are focusing on the development of artificial BMs (ABMs). ABMs are acoustic sensors that mimic the tonotopy of the cochlea: passive frequency selectivity and acoustic-to-electric energy conversion. The frequency selectivity of ABMs has been realized by varying the beam length^8^,^9^,^10^,^11^,^12^,^13^, membrane width^5^,^14^,^15^,^16^,^17^,^18^,^19^, and beam thickness^20^. The energy conversion of the cochlea can be achieved by a piezoelectric effect^8^,^9^,^14^,^15^,^19^,^21^, piezoresistive effect^22^,^23^,^24^, and optical readout^12^,^17^.

Beam length is an important ABM design parameter for mimicking the frequency selectivity of cochleae. The resonance frequencies of a beam array vary depending on the length of the beams. Tanaka *et al.*^11^ and Xu *et al.*^12^ proposed a micro-cantilever array that mimics the mechanical performance of the BM. Another parameter that can be varied to mimic the appropriate frequency selectivity is the membrane width of an ABM. Zhou *et al.*^17^ and Wittbrodt *et al.*^18^ demonstrated a membrane-type ABM consisting of two fluid-filled chambers separated by a polymer membrane. The width of the membrane varied along the length of these chambers; a narrow membrane vibrates at high frequency, while a wide membrane senses low frequency. White and Grosh fabricated a microelectromechanical system (MEMS)-based membrane-type ABM^5^. Recently, Shintaku *et al.* proposed a microbeam array ABM that uses thickness as the variable parameter^20^.

Among the above-mentioned transduction methods, the piezoelectric effect has an advantage for realizing an ABM, because it directly converts acoustic energy into electrical energy with no need for an additional energy source. Several piezoelectric materials have been used to fabricate ABMs. Chen *et al.*^14^ and Shintaku *et al.*^15^ proposed a membrane-type ABM using polyvinylidene fluoride (PVDF). PVDF is a flexible piezoelectric polymer suitable for membrane-type ABMs. Lee *et al.* reported a highly efficient ABM using a Pb[Zr_*x*_ Ti _1− *x*_]O_3_ (PZT) thin film on a flexible silicone-based membrane^25^. Aluminum nitride (AlN) is a good candidate material for fabrication of ABMs, because it allows the size of micromachined devices to be reduced drastically while preserving good performance^26^. Also, an AlN ABM can be integrated with a silicon complementary metal-oxide-semiconductor (CMOS) chip to achieve a miniaturized system.

Cochlear implants (CIs) are sensorineural prosthetic devices that provide a sense of sound to patients with severe-to-profound sensorineural hearing loss (SHL) by directly stimulating the auditory neurons in recipients who can no longer hear speech clearly using hearing aids. As mammalian hair cells do not regenerate spontaneously^27^,^28^, CIs are the optimum therapeutic solution for SHL in which loss of hair cells is the predominant pathology^29^. Although the performance of CIs is acceptable, the requirement to wear an external processor that communicates with the implanted device via a radio-frequency coil increases the power consumption, limits the activities that can be undertaken while wearing the device, leads to some patients feeling stigmatized and contributes to the high cost of these devices^30^,^31^,^32^,^33^. To overcome these limitations, researchers have attempted to develop a next-generation CI that integrated a microfabricated ABM^8^,^9^,^15^,^16^,^25^,^33^,^34^.

Bachman *et al.* proposed an ABM using an optical readout for a bionic ear. Although an optical readout has an advantage in terms of power consumption, energy is required to realize tonotopy using a laser diode and an optical detector, which could result in a relatively bulky device^33^. Shintaku *et al.* reported a membrane-type ABM for an implantable self-powered device using PVDF^15^. Subsequently, Inaoka *et al.* showed electrically evoked auditory brainstem responses (EABRs) using a membrane-type ABM^16^. Although the membrane-type ABM successfully induced a biological auditory brainstem response, it showed limited frequency selectivity. In our previous study, we compared the frequency selectivity of a membrane-type ABM and a beam array-type ABM^35^. The results showed that the frequency selectivity of our membrane-type ABM was poor because of the mechanical coupling of the membrane among the sensing channels on the same membrane. Shintaku *et al.* also reported the measurement of EABRs to evaluate effect of the frequency selectivity in membrane type ABM^36^. But the unclear frequency selectivity of membrane-type ABM was shown in the measurement of the EABRs.

In this work, a novel ABM was fabricated using a MEMS-based piezoelectric cantilever array to realize a clear tonotopy without the need for an external battery. Frequency selectivity and sensitivity to incoming sound pressure were estimated. Also, an animal model was used to verify the characteristics of the ABM as a possible front end for future CI applications. For this, a signal processor was used to convert the piezoelectric output from the ABM to an electrical signal to stimulate the auditory neurons in the cochlea. The results demonstrated that the ABM with a signal processor and intra-cochlear electrode array could induce an auditory evoked potential from deafened guinea pigs, with clear frequency selectivity by way of an implanted intra-cochlear electrode array.

## Results

### Realization of an ABM using a MEMS cantilever array

**Figure 1:**
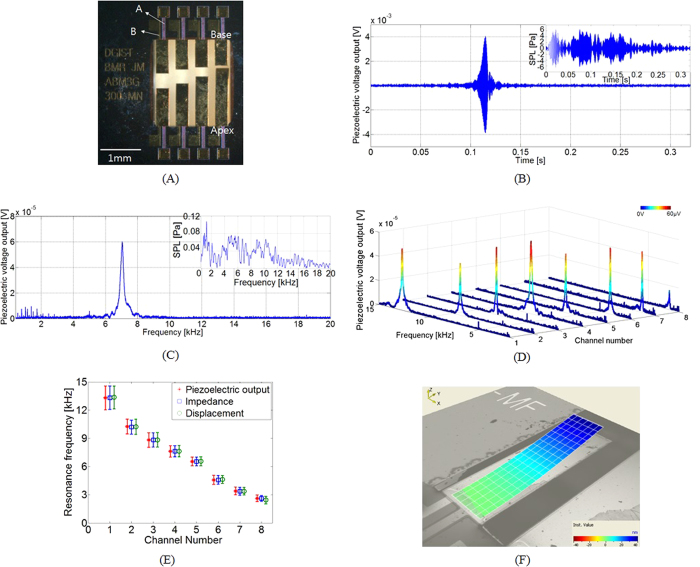
(**A**) Optical image of the fabricated ABM, which consists of an array of eight cantilevers. The longest and shortest beams correspond to the apical and basal regions of the cochlea, respectively. A: Bottom electrodes (Mo/Ti). B: Top electrodes (Au/Ti). Aluminum nitride (AlN) was used as the active piezoelectric layer between the two electrodes. (**B**) Piezoelectric output voltage during the application of acoustic stimuli to the fourth channel of an ABM. The input acoustic stimuli are shown in the upper right corner. (**C**) Frequency response of the piezoelectric output voltage measured in Fig. 1B. The frequency responses of the input acoustic stimuli are shown in the upper right corner. (**D**) Piezoelectric voltage outputs for all cantilevers of an ABM, demonstrating not only the piezoelectric characteristics but also clear frequency selectivity. (**E**) Mean values of the resonance frequencies of the cantilevers of four ABMs measured by piezoelectric voltage output, electrical impedance, and mechanical displacement. (**F**) A deformed cantilever during the vibration at the corresponding resonance frequency measured using a scanning laser Doppler vibrometer.

The length of cantilevers is a design parameter used to mimic the frequency selectivity of the BM. The fabricated ABM is composed of eight piezoelectric cantilevers (Fig. 1A). The longest cantilever was located opposite the shortest cantilever to minimize the total size of the device. The width of the cantilevers was 300 μm, and their lengths varied from 600 to 1350 μm (Table 1). The total volume of the ABM was 2.5 × 2.5 × 0.6 mm^3^, which is sufficiently small for implantation in the external auditory meatus or tympanic membrane^37^,^38^. A double-sided polished p-type (100) silicon-on-insulator (SOI) wafer with a diameter of 15.2 cm was used for device fabrication. The wafer was composed of a 2-μm Si device layer, a 1-μm SiO_2_ buried oxide (BOX) layer, and a 600-μm Si handle layer. The AlN thin film was used as the active piezoelectric layer and was sandwiched between a molybdenum (Mo) layer and a gold (Au) layer as the bottom and top electrodes, respectively. Reactive ion etching (RIE) and deep RIE (DRIE) were used to release the freestanding cantilever array. All fabrication steps used in this study were compatible with batch processes. The details of the fabrication process are described in Fig. S1 and in the Methods.

### Characterization of the tonotopy of the ABM

A periodic chirp signal with a frequency range of 42 Hz to 20 kHz was used to vibrate the cantilevers in an anechoic chamber. The overall sound pressure level (SPL) during the characterization of the ABM was ca. 101.7 decibel SPL (dB SPL). The piezoelectric output from the fourth channel of an ABM is shown in Fig. 1B; the applied acoustic stimuli are shown inset. Figure 1C shows the frequency response of the piezoelectric output voltage measured in Fig. 1B. The largest peak at 7.04 kHz indicates the resonance frequency of the corresponding cantilever. The strength of the piezoelectric output voltage from the fourth channel of the ABM was 4.06 mV at the resonance. The maximum sensitivity of the fourth channel was 1.67 mV/Pa, and the sensitivity of the ABM was in the range of 0.354–1.67 mV/Pa (Table 1). These represent an improvement over our previously reported values of 0.114–0.48 mV/Pa at the resonance frequency^9^. Also, these results show that the fabricated ABM could realize an acoustic-to-electric energy conversion, with a self-powered sensing capability.

Figure 1D presents the piezoelectric outputs of the ABM in a frequency domain to demonstrate the resonance frequencies of the cantilever array. The frequency selectivity is evident in the frequency range of 2.92–12.6 kHz: a lower channel number indicates a shorter cantilever length, which causes a higher resonance frequency. The resonance frequencies of each cantilever consistently shifted to lower frequencies as the channel number increased (i.e., with increasing cantilever length). The quality factors of each cantilever at its resonance frequency are shown in Table 1. In addition, the mean values of the resonance frequencies of the four ABMs were measured by piezoelectric output, electrical impedance^39^, and mechanical displacement, as shown in Fig. 1E. A vibration pattern at a resonance frequency is also shown in Fig. 1F. The resonance frequencies measured by three different methods were in good agreement and are presented in Table 1. The results demonstrated that the eight channels of the fabricated ABMs have clear frequency selectivity and showed potential for mimicking the tonotopy of the BM.

### Use of a signal processor for conversion of the piezoelectric outputs into electrical signals to stimulate auditory neurons

**Figure 2:**
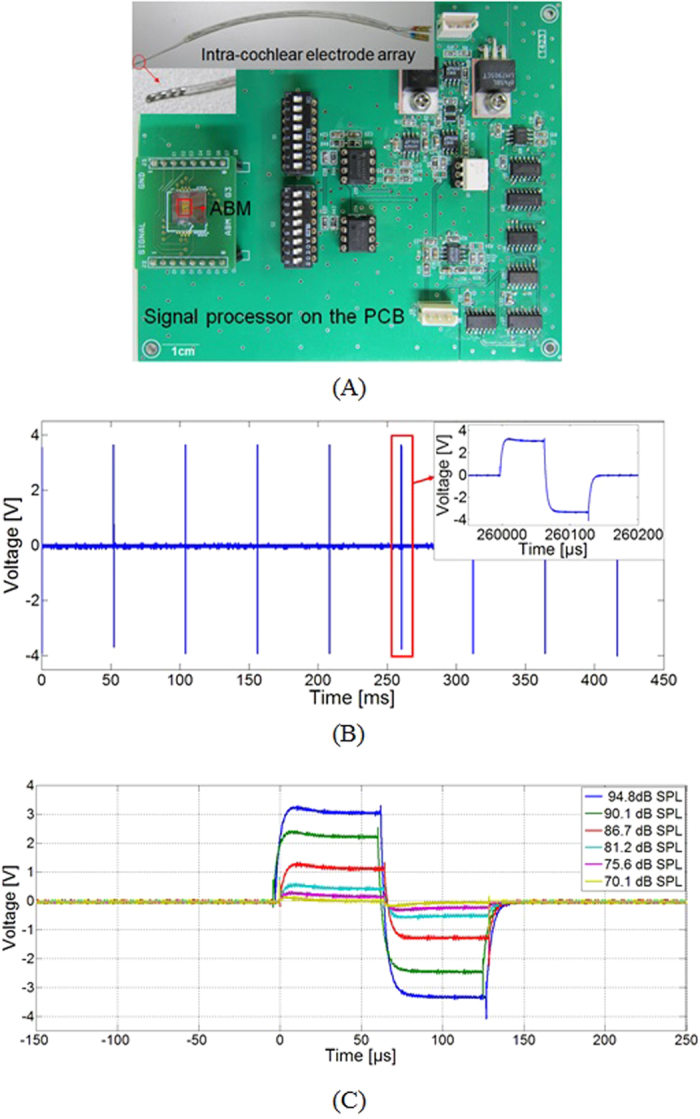
(**A**) A photograph of the proposed ABM, a signal processor, and an intra-cochlear electrode array. (**B**) A 19.8-Hz biphasic stimulating electrical signal with a 65-μs pulse-width was measured at the output module of the signal processor during application of acoustic stimuli of 94.8 dB SPL to the ABM. (**C**) The amplitude was modulated depending on the applied SPL (70.1–94.8 dB SPL).

As shown in Fig. 2A, the ABM incorporated a signal processor to convert acoustic stimuli into electrical signals with frequency selectivity. The circuit for the signal processor was fabricated on a printed circuit board to generate electrical stimulation signals based on the piezoelectric outputs of the ABM initiated by the applied acoustic pressure. An on-and-off switch can control individual ABM channels, and the piezoelectric signal from the ABM was amplified 950-fold using an operational amplifier (op-amp). A low-noise CMOS op-amp LTC6241 (Linear Technologies, USA) was used because the electrical impedance of the piezoelectric cantilever is relatively high (255.4–730.7 kΩ)^40^. The amplified signal was then converted to DC voltage using a rectifier circuit, and this signal was used as a reference voltage to determine the magnitude of the signal required to stimulate auditory neurons. A logic circuit with a clock pulse generator was used to control pulse width and the period of a charge-balanced biphasic square wave. The amplitude of biphasic signals was modulated depending on the amplitude of the reference voltage, which is a function of the applied sound pressure. Therefore, the amplitude of the stimulating signal could be controlled using the input SPL to the ABM.

The sixth channel of the ABM was turned on, and an input sound was applied at the resonance frequency of the cantilever (Fig. 2B). The figure shows the waveforms of the stimulating electrical signals from the signal processor by the pure-tone acoustic stimuli of 94.8 dB SPL at 4.43 kHz, which is the resonance frequency of the sixth channel of the ABM. Based on the piezoelectric output of the ABM, the signal processor generated an electrical stimulation signal for auditory neurons at 19.8 Hz. The stimulation signal is a charge-balanced biphasic signal with a 65-μs pulse-width to stimulate auditory neurons. The amplitude of the stimulating signal was modulated from 0.15 V to 3.5 V, corresponding to the input SPL from 70.1 to 94.8 dB SPL at 4.43 kHz, as shown in Fig. 2C.

### Verification of the performance of ABM as a front end for potential cochlear implant applications using an animal model

**Figure 3:**
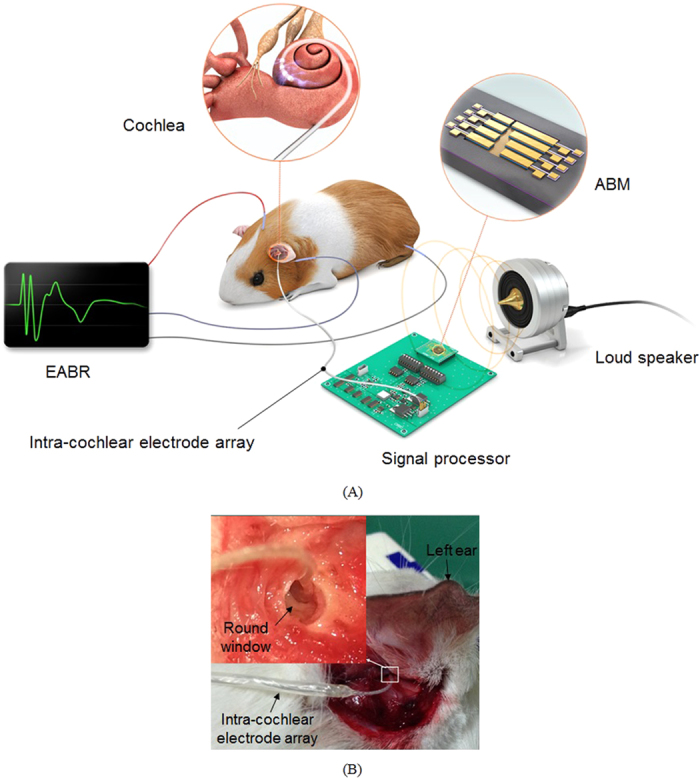
(**A**) Schematic view of an experimental setup with the ABM. The fabricated ABM was assembled incorporating a signal processor and an intra-cochlear electrode array. When applying acoustic sounds to the ABM, a cantilever in the ABM generates a piezoelectric output, which is converted by the signal processor into a stimulating electrical signal. The output of the signal processor is linked to the intra-cochlear electrode array inserted into the cochlea of a deafened guinea pig. Finally, EABRs in response to the electrical stimulation of the auditory neurons in the brain of the deafened guinea pig are recorded. (**B**) The intra-cochlear electrode array inserted through the round window of the guinea pig.

A schematic view of the experimental setup is shown in Fig. 3A. When an acoustic stimulus of a certain frequency is applied to the ABM by a loudspeaker, one piezoelectric cantilever with a resonance frequency close to the applied frequency vibrates and generates a piezoelectric signal. The ABM was mounted on the signal processor to facilitate transfer of the piezoelectric output. A custom-built intra-cochlear electrode array^41^ was linked to the output port of the signal processor to stimulate the auditory neurons of the deafened guinea pig. The intra-cochlear electrode array was then inserted through the round window into the scala tympani of the cochlea (Fig. 3B). Immediately following the insertion, an acute study was undertaken in deafened guinea pigs to evaluate EABR responses to acute electrical stimulation; for this purpose, a recording system with needle electrodes in the scalp was used.

**Figure 4:**
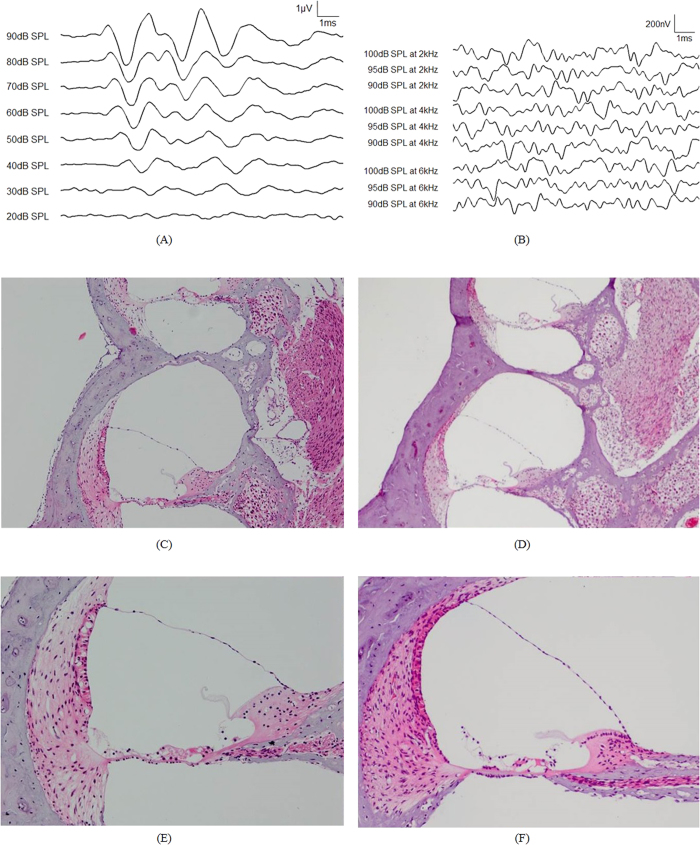
(**A**) ABRs induced in a guinea pig by acoustic click stimuli at 20–90 dB SPL before deafening. (**B**) ABRs induced in a deafened guinea pig by application of tone-burst stimuli at 90–100 dB SPL at 2, 4, and 6 kHz. Only noise signals were evident after deafening. Histology of cochleae in guinea pigs with deafness following application of kanamycin and furosemide (**C** and **E**) and with normal hearing (**D** and **F**). H&E staining of cochlear sections showed no marked degeneration in spiral ganglion neurons between cochleae (**C** and **D**) (100 × objective lens). All hair cells are absent in the cochlea of deaf guinea pigs compared with those with normal hearing (**E** and **F**) (200 × objective lens).

We recorded acute electrophysiological EABRs from six ears of deafened Hartley guinea pigs (five males; both ears of one animal were used). Before deafening, click-evoked auditory brainstem responses (ABRs) were used to identify the normal hearing of guinea pigs (Fig. 4A). Ototoxic deafening was then performed to reduce the electrophonic activity in the EABR. Kanamycin (80 mg/100 g; subcutaneous injection) and furosemide (15 mg/100 g; intraperitoneal injection) were administered to the guinea pigs. A tone-burst-evoked ABR was used to confirm complete hearing loss of the guinea pigs at 7 days after drug administration (Fig. 4B). The histological data verified a severe loss of hair cells and survival of spiral ganglion neurons (SGNs)^42^. Hematoxylin and eosin (H&E) staining was conducted to assess cochlear histology in the deafened (Fig. 4C) and normal (Fig. 4D) guinea pigs. There was no significant degeneration of SGNs in both cases. Figure 4E shows loss of hair cells in a deafened guinea pig, while Fig. 4F shows normal hair cells. Figure 4E,F are higher-magnification versions of the images of a deafened and normal guinea pigs shown in Fig. 4C,D, respectively.

### EABR using an ABM with a signal processor

**Figure 5:**
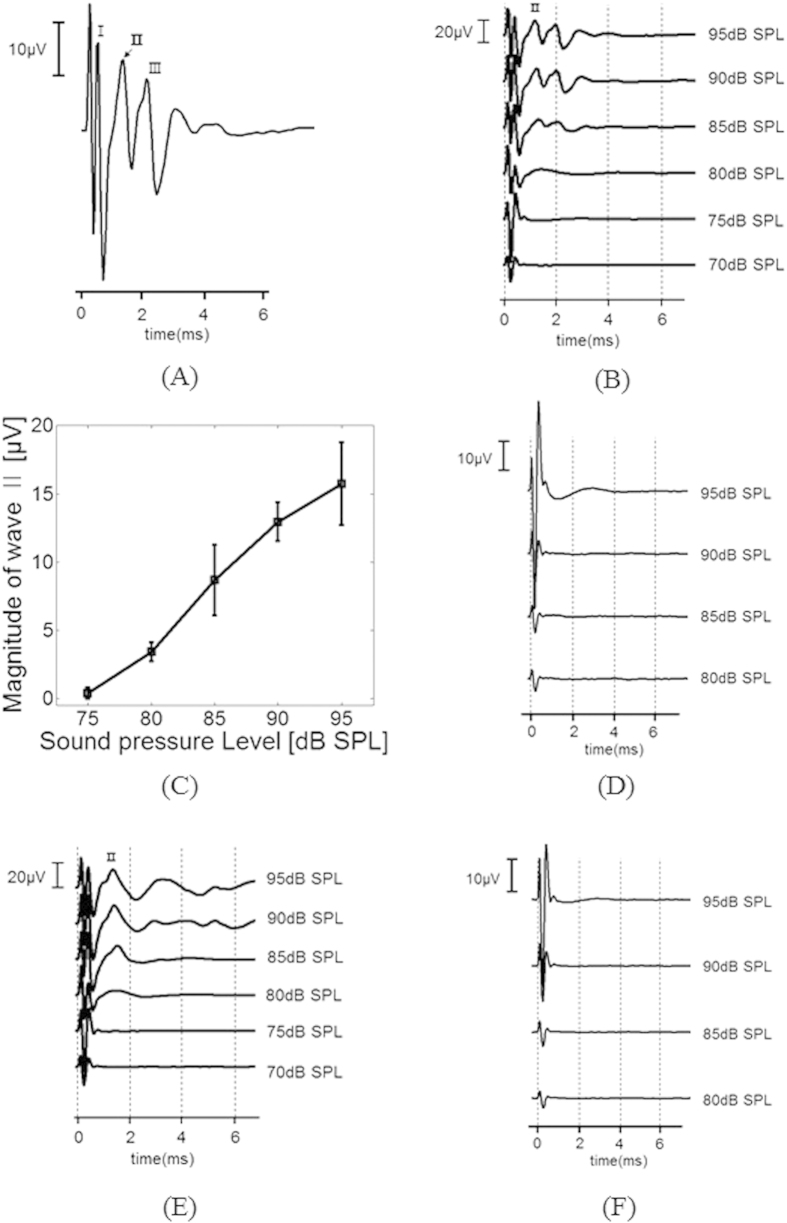
(**A**) Recorded EABRs evoked by stimulation of the auditory neurons using electrical signals from the sixth channel of the ABM with pure-tone acoustic stimuli of 95-dB SPL at 4.43 kHz, which is the corresponding resonance frequency. Stimulus onset was at time 0. Waves I, II, and III are indicated in the EABR recordings. (**B**) Recording of EABRs with acoustic stimuli from 70–95-dB SPL at 4.43 kHz to the same channel as in (**A**). (**C**) The magnitude of wave II as a function of the input sound pressure. (**D**) Recordings of EABRs with acoustic stimuli from 80 to 95 dB SPL at the sixth channel of the ABM with pure-tone acoustic stimuli at 6.85 kHz, which is an off-resonance frequency of the cantilever. (**E**) Recordings of EABRs using the fourth channel of the ABM with pure-tone acoustic stimuli at 6.85 kHz, which is the resonance frequency of the corresponding cantilever. (**F**) Recordings of EABRs using the fourth channel of the ABM with pure-tone acoustic stimuli at 4.43 kHz, which is an off-resonance frequency of the cantilever.

Figure 5A shows the EABR with a sound input of 95 dB at 4.43 kHz, which is the resonance frequency of the sixth channel of the ABM. A large stimulus artifact in the EABR recording occurred before the first wave. The other waveforms of the EABR recordings were consistent with those reported previously^43^,^44^. The first wave could also be obscured by electrical artifacts, and wave III could be influenced by the digastric muscle response^45^. Therefore, we analyzed wave II to determine the threshold of EABRs^43^. Figure 5B shows the EABR recordings with acoustic stimuli from 70 to 95 dB SPL at 4.43 kHz. The latency of wave II was identified at 1.26 ± 0.14 ms in the test animals. The threshold of wave II was 75 dB SPL. Figure 5C shows the magnitude of wave II as a function of the input SPL. The magnitude of wave II increased with the level of input sound pressure applied to the ABM. Although the sensitivity to sound was still lower than the threshold of human hearing, these EABR recordings clearly demonstrate that the proposed ABM incorporating a signal processor could electrically stimulate auditory neurons due to the input acoustic stimuli.

The frequency selectivity of ABM was assessed by measuring EABR magnitude while opening a particular channel of the ABM. The opened ABM channel generates piezoelectric output only if the input frequency matches the resonance frequency of the corresponding piezoelectric cantilever. The EABRs were measured using the sixth channel of the ABM while applying a pure-tone sound at 6.85 kHz, which is an off-resonance frequency of the sixth channel. As shown in Fig. 5D, only electrical artifacts resulted when an off-resonance frequency of the corresponding cantilever was applied. Because it is insensitive to off-resonance frequencies, the cantilever cannot generate sufficient piezoelectric output to initiate the electrical stimulation signals from the signal processor. For further confirmation of the frequency selectivity, the fourth channel of the ABM was subjected to the same experiment with acoustic stimuli at 6.85 kHz (the resonance frequency) and 4.43 kHz (an off-resonance frequency). The data in Fig. 5E,F confirm the frequency selectivity of ABM, as determined from the EABR measurements.

## Discussion

A novel ABM based on a piezoelectric MEMS cantilever array was developed for use as the front end of a cochlear implant. Our previously reported ABMs were based on a beam array to enable clear separation of frequency^8^,^9^,^10^. The frequency selectivity of our previous ABMs was identified by beam displacement and piezoelectric output voltage during application of sound pressure. The response frequency range was 10.2–34.3 kHz, and the sensitivity was 0.114–0.48 mV/Pa^9^. In this work, we selected a piezoelectric cantilever array ABM to lower the response frequency range, to improve the sensitivity, and to minimize the device footprint. The fabricated piezoelectric cantilever array ABM demonstrated a clear frequency selectivity in the range of 2.92–12.6 kHz using eight cantilevers. The piezoelectric output signal was in the range of 0.354–1.67 mV/Pa for the corresponding sound frequency of 42 Hz to 20 kHz at 101.7 dB SPL. Although the frequency range does not yet cover the standard range of audible frequencies, we successfully demonstrated the possibility of using the piezoelectric cantilever array ABM for frequency separation. While the sensitivity of the current ABM was improved markedly compared with our previous ABMs, there remains room for further improvement. Further research should focus on lowering the response frequency range and increasing the sensitivity. The possible approaches include optimization of the material or adaptation of the design of a new device^46^,^47^,^48^.

Several researchers have attempted to develop various types of ABM for CI applications^5^,^11^,^12^,^13^,^14^,^17^,^18^,^19^,^25^,^33^,^34^. The majority of previous studies focused only on ABM^10^,^11^,^12^,^13^,^16^,^17^,^18^,^19^,^25^ itself or simply proposed the concept of CI^33^,^34^; Inaoka *et al.* were the first to conduct animal testing of an ABM^16^. In the work cited by Ref. 16, the membrane-type ABM that was reported by Shintaku *et al.* was used^15^. Although this research is promising, the frequency selectivity of the membrane-type ABM was insufficient. Similar results were also reported by a study on the frequency selectivity of an AlN-membrane-type ABM and an AlN-beam array-type ABM^35^. Based on this comparison, the proposed cantilever-array ABM was selected because of the clear frequency selectivity. The proposed cantilever-array ABM showed a higher quality factor (43.7–133) in frequency response than did the ABM reported by Shintaku *et al.* (5.51–12.3)^15^,^49^. This indicates that the proposed cantilever array-type ABM can separate frequency with higher resolution. In terms of the device size, the ABM reported by Shintaku *et al.* is on a centimeter scale (4.7 × 1.7 cm), while the proposed cantilever-array ABM is on a millimeter scale (2.5 × 2.5 mm). Although packing must be considered for an actual implantable artificial cochlea, a smaller device would confer several advantages for clinical applications.

Inaoka *et al.* reported the recording of EABRs using a membrane-type ABM and demonstrated that their device could induce an auditory evoked potential in deafened guinea pigs. Also, Shintaku *et al.* evaluated the frequency selectivity by showing variations in the magnitude of the EABRs^36^. When a sound pressure with a particular frequency was applied to the membrane-type ABM, the channel corresponding to that sound frequency responded by generating EABR; however, the adjacent channels also generated EABRs. This resulted in unclear frequency selectivity of the membrane-type ABM. In contrast, the proposed cantilever-array ABM showed clear frequency selectivity. When we opened a channel in our proposed ABM, we applied two different sound frequencies: the resonance frequency and an off-resonance frequency of the corresponding cantilever of the selected channel. In this case, EABRs were measured only when the resonance frequency was applied. Therefore, the proposed cantilever-type ABM showed clear frequency selectivity.

In addition, Inaoka *et al.* proposed a life-sized device designed to operate in the cochlea and to exploit the vibration of the BM^16^. In this approach, the device was operated by hydromechanical stimulation in the cochlea by placing the membrane-type ABM on the BM. However, it would be challenging, if not impossible, to implant an artificial membrane-type ABM in the basilar turn of the human cochlea, which is filled with fluid. Even if the device can be implanted at the necessary position, the immune response may cause fouling around the ABM and inhibit the vibration of the device. Therefore, a different approach was used for the proposed cantilever-array ABM such that it could be implanted into the external auditory meatus or near the eardrum to enable direct sensing of acoustic stimuli.

Although the signal processor currently incorporated with the ABM is not sufficiently small for implantation, further miniaturization could be accomplished by integrating MEMS and a CMOS^50^ as the ABM and the signal processor, respectively. The total volume of any future stimulation system using an ABM will be drastically reduced by integration of MEMS-CMOS including a state-of-art intra-cochlear electrode array^51^. Therefore, we envision the development of a low-cost MEMS-CMOS integrated CI containing an ABM, a signal processor, and an intra-cochlear electrode array for a next-generation CI by low-cost batch fabrication. Although the proposed ABM operates without external energy, the signal processor requires energy to generate electrical signals for stimulating auditory neurons. A fascinating study recently reported energy harvesting using the endocochlear potential and the motions of the heart, lung, and diaphragm^52^,^53^. If this type of biological energy harvester can supply sufficient energy for the stimulation system, a self-powered next-generation CI may be possible, and many limitations of current CIs could be overcome.

## Methods

### Fabrication of an artificial basilar membrane

For fabrication, 15.2-cm diameter *p*-type (100) SOI wafers with a 2-μm Si device layer, 1-μm buried oxide, and 600-μm handle layer were used (Fig. S1A); a flow diagram of the fabrication process is provided in Fig. S1. Wet oxidation was performed to grow a 200-nm-thick SiO_2_ layer to serve as a dielectric layer between the top silicon layer and the bottom electrode (Fig. S1B). A 20-nm titanium (Ti) and a 180-nm molybdenum (Mo) layer were deposited by DC sputtering to serve as the bottom electrode. The Mo and Ti layers were patterned using aluminum (Al) etchant type-A by photolithography (Fig. S1C). A 500-nm piezoelectric AlN layer was deposited by sputtering (Plainview, Veeco Instruments Inc., USA) on the Mo layer. The sputtered AlN was patterned by photolithography using AZ 7220 photoresist and CD30 developer. RIE was then conducted using a mixed BCl_3_ and Cl_2_ gas (Fig. S1D). Photolithography for patterning the top electrode was performed using the lift-off process. AZ 5214 negative photoresist and CD30 developer were used to create the top electrode pattern. After patterning of AZ 5214, a 20-nm Ti adhesion layer and a 180-nm layer of gold (Au) were deposited by an evaporator (BJD 2000, Temescal, USA). After removing the sacrificial AZ 5214, the top electrode was formed on the AlN layer (Fig. S1E). In addition, a photolithography process was used to pattern the cantilevers, and the exposed SiO_2_ layer was etched away using RIE (Fig. S1F). The exposed device silicon layer was etched away by DRIE (Fig. S1G). To realize a freestanding cantilever array, the handle silicon layer on the backside must be etched by DRIE. Prior to the DRIE process, an Al mask layer was sputtered on the backside of the wafer and patterned for DRIE because a polymer-based photoresist may cause burning during DRIE. After patterning the Al layer, the exposed part of the SiO_2_ layer was etched away by RIE. DRIE was then performed to remove the silicon beneath the buried oxide layer, as shown in Fig. S1H. Finally, the box layer under the cantilevers was etched away using buffered oxide etcher (BOE) to release the cantilevers (Fig. S1I).

### Characterization of the artificial basilar membrane

The piezoelectric outputs and displacements of the cantilevers were measured in a custom-made anechoic chamber (background noise: 18.7 dBA). The measurements were obtained using an MSA-500 micro system analyzer (Polytec GmbH, Germany) integrated with a scanning laser Doppler vibrometer (SLDV). The periodic chirp signal from the junction box of SLDV was applied to the device using a loudspeaker (A7X, Adam, Germany) that was vertically located 50 cm from the ABM and inclined at an angle of approximately 40 degrees with respect to the plane of the ABM. A reference microphone (4189, Bruel and Kjaer, Germany) and sound pressure meter (2239A, Bruel and Kjaer, Germany) were placed near the test sample to measure the acoustic stimulus. The sampling frequency and the sampling time of the measurements were set to 2.56 MHz and 6.4 ms, respectively. The resonance frequencies of all cantilevers in air were also measured using an impedance analyzer (4294A, Agilent Technology, USA).

### Characterization of the signal processor incorporated into the ABM

The environment used for characterization of the signal processor incorporated in the ABM was identical to that used to characterize the ABMs. The stimulating electrical signals from the signal processor were measured to estimate the sensitivity of the ABM. Pure-tone acoustic stimuli at 4.43 kHz were applied to ABM using a loudspeaker (A7X, Adam, Germany), and the output signals at the signal processor module were measured using an oscilloscope (DSO-X 2004A, Agilent Technology, USA). The SPL was also measured using a sound-level meter (2239A, Bruel and Kjaer, Germany) placed next to the ABM to obtain reference data. The acoustic stimuli were varied over the range of 70.1–94.8 dB SPL in descending steps of ~5 dB SPL.

### Experimental animals and ethics

Six ears of Hartley guinea pigs (five males, both ears of one animal were used) weighing 150–200 g were used to record EABRs to characterize the performance of the proposed ABM. The animals were anesthetized using mixtures of tiletamine–zolazepam (1.8 mg/100 g) and xylazine hydrochloride (0.7 mg/100 g) administered intramuscularly. All animals and experimental protocols were approved by the Institutional Review Board of Kyungpook National University Hospital (approval number, KNU 2014-12). All procedures were performed on an anti-vibration table (VH3048W-opt, Newport Co., USA) in a soundproof room.

### ABR recording

Auditory brainstem responses (ABRs) were recorded in a soundproof and electrically shielded room. The stimulating and recording system was a Tucker Davis Technologies System 3 (RZ6, Tucker Davis Technologies Inc. (TDT), USA) coupled with the BioSigRZ software (TDT, USA). The parameters of BioSigRZ were as follows: a 24.4-kHz sampling rate, 24-bit sigma-delta of the analog-to-digital converter, 300–3,000-Hz filter, 60-Hz notch, and 20× gain. Acoustic stimuli were delivered through a loudspeaker (MF1, TDT, USA) placed 10 cm from the applied pinna; the opposite pinna was occluded with Otoform (Dreve, Unna, Germany). A click signal and tone-burst signal consisting of five-cycle sinusoids (rise/fall two cycles and plateau one cycle) were used as the acoustic stimuli and were generated by the software. Both acoustic stimuli were generated every 36.1 ms for 500 repetitions. To reduce artifacts due to repetitive stimuli, the phase of each stimulus was reversed. The click signal was generated from 90 dB SPL in descending steps of 10 dB SPL to confirm normal hearing in the guinea pigs prior to deafening. To confirm deafness in the guinea pigs, we used a tone-burst signal from 100 dB SPL in descending steps of 5 dB SPL at 2, 4, and 6 kHz. Needle electrodes were used to measure the ABRs. The needle electrodes from the head stage (RA4LI, TDT, USA) connected to a pre-amplifier (RA4PA, TDT, USA) were inserted in the vertex (non-inverting electrode), mastoid (inverting electrode) and hind leg (ground). The RA4PA with RA4LI had a gain of 20× and frequency responses of 2–7500 Hz. The impedances were tested by the head stage and were less than 1 kΩ.

### Animal surgery

A post-auricular incision was made, and the overlying muscle tissue was dissected to expose the posterior bulla. A hole was drilled through the bulla to expose the round window. A linear incision was made in the round-window membrane using a pick and a custom-designed intra-cochlear electrode array was inserted into the scala tympani. The intra-cochlear electrode array consisted of four ring-type active platinum electrodes with a width of 0.3 mm in a silastic carrier with a 0.75-mm center-to-center distance between adjacent electrodes^41^. The electrical outputs from the signal processor were wired to the intra-cochlear electrode array, which delivered the electrical stimuli through a bipolar configuration between the basal ring (the fourth) and apical (the first) electrodes of the intra-cochlear electrode array.

### EABR recording

EABRs were recorded in a manner similar to the ABR measurements. For EABR measurements, the recording was triggered by the signal from the signal processor (5 V transistor–transistor logic pulse, 19.8 Hz). An input port was used to deliver an external trigger, and the acquisition time was set using RpvdsEX software (TDT, USA). Although the trigger signal was generated simultaneously with the stimulating signal, there was an unexpected delay of 2–3 ms because of the processing time in the TDT system 3. Therefore, the exact acquisition timing was delayed by 47 ms (2–3-ms delay from the first period of the stimulating signal) to record the second stimulation signal among the 500 repetitions. Thus, the stimulus onset was at time 0. The data acquisition digitization parameters were identical to the ABR recording parameters (i.e., a 24.4-kHz sampling rate, 24-bit sigma-delta of the analog-to-digital converter, 300–3,000-Hz filter, 60-Hz notch, and 20× gain).

## Additional Information

**How to cite this article**: Jang, J. *et al.* A microelectromechanical system artificial basilar membrane based on a piezoelectric cantilever array and its characterization using an animal model. *Sci. Rep.* **5**, 12447; doi: 10.1038/srep12447 (2015).

## Supplementary Material

Supplementary Information

## Figures and Tables

**Table 1:** ABM lengths, sensitivities, bandwidths, and quality factors, and the resonance frequencies of the cantilevers measured by means of piezoelectric voltage output, electrical impedance, and displacement. The resonance frequencies are average values of four ABMs.

**ABM channel number**	**Cantilever length (μm)**	**Sensitivity to sound [mV/Pa]**	**Bandwidth (Hz)**	**Quality factor (Q-factor)**	**Average resonance frequencies (kHz)**		
					**Piezoelectric**	**Impedance**	**Displacement**
1	600	1.22	104	121	13.3	13.3	13.4
2	640	0.859	69.4	134	10.3	10.2	10.2
3	690	0.967	42.5	189	8.83	8.84	8.84
4	780	1.67	114	61.7	7.62	7.63	7.63
5	880	0.890	51.8	116	6.54	6.55	6.56
6	990	0.708	52.3	95.2	4.59	4.59	4.63
7	1150	0.753	36.3	88.4	3.41	3.38	3.38
8	1350	0.354	66.8	43.7	2.63	2.62	2.44
