# Supplementary material for: A microelectromechanical system artificial basilar membrane based on a piezoelectric cantilever array and its characterization using an animal model
Source: Sci Rep. 2015 Jul 31;5:12447. doi: 10.1038/srep12447 (PMC4521187; doi:10.1038/srep12447)
Supplement: Supplementary Information [file srep12447-s1.doc]

Supplementary Information

Manuscript title: A microelectromechanical system artificial basilar membrane based on a piezoelectric cantilever array and its characterization using an animal model

Authors: Jongmoon Jang, JangWoo Lee, Seongyong Woo, David J. Sly, Luke J. Campbell, Jin-Ho Cho, Stephen J. O’Leary, Min-Hyun Park, Sungmin Han, Ji-Wong Choi, Jeong Hun Jang, and Hongsoo Choi


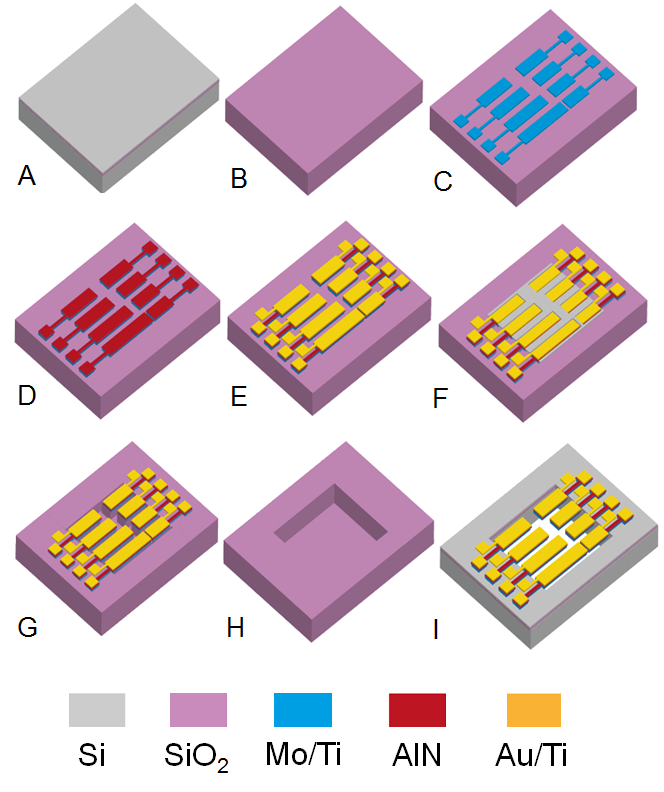


Fig. S1. Schematic view of the fabrication process for the proposed ABM. (A) SOI wafers with a 2-μm Si device layer, 1-μm buried oxide, and 600-μm handle layer; (B) wet oxidation of a 200-nm SiO_2_ layer; (C) bottom electrode (a 20-nm titanium (Ti) and a 180-nm molybdenum (Mo) layer); (D) piezoelectric layer (a 500-nm aluminum nitride (AlN) layer); (E) top electrode (a 20-nm Ti and a 180-nm layer of gold (Au)); (F) reactive ion etching (RIE); (G) deep reactive ion etching (DRIE) for patterning of the cantilever array; (H) DRIE of the backside to etch away the handle layer to generate a free-standing cantilever array; and (I) wet etching of the box layer by a buffered oxide etcher (BOE)
